# Resting-state Fronto-limbic Connectivity in Unipolar Depressive Patients as a Predictor for Sporadic Conversion to (Hypo)mania

**DOI:** 10.1192/j.eurpsy.2023.1462

**Published:** 2023-07-19

**Authors:** I. Tajioui, T. van Neerven, M. J. van Tol, J. M. Bas-Hoogendam, D. Veltman, N. van der Wee, M. de Leeuw

**Affiliations:** 1 Psychiatry, Leiden University Medical Center; 2 Leiden Institute for Brain and Cognition, Leiden; 3 Neuroscience, University Medical Center Groningen, Groningen; 4 Developmental and Educational Psychology, Leiden University, Leiden; 5 Psychiatry, Amsterdam University Medical Center, Amsterdam, Netherlands

## Abstract

**Introduction:**

Patients with a bipolar disorder (BD) may experience one or several episodes of major depression before transitioning into a manic phase. Given that treatment with common antidepressants may exacerbate symptoms of mania in patients with BD, initial diagnosis of major depressive disorder (MDD) is a significant issue affecting BD patients. Currently, a family history of BD is used as an early identifier for BD, but as genetic factors associated with BD confer susceptibility to a wide range of psychiatric illnesses, this approach lacks specificity. Thus, there is a pressing need for a biomarker which specifically predicts development of bipolar symptoms in patients with MDD, rather than a trait vulnerability to psychiatric disorder

**Objectives:**

The aim of this study is to assess alterations in fronto-limbic function that exist prior to the manifestation of the first manic episode in BD patients without familial predisposition for BD.

**Methods:**

To identify a biomarker for conversion to BD we performed a study in a naturalistic sample of MDD patients without a familial risk for BD, which were followed for 9 years. We used a seed-based functional connectivity analysis to assess differences in resting-state fronto-limbic connectivity between MDD patients who converted to BD during the follow-up period (n = 11), and non-converting MDD patients (n = 56).

**Results:**

Clusters of significantly reduced functional connectivity were found in the fronto-limbic network of prospective converters relative to non-converters.

**Conclusions:**

Findings suggest that alterations of fronto-limbic functional connectivity during episodes of depression predate and associate with conversion to BD later in life, in the absence of familial risk. These fronto-limbic functional connectivity disturbances may be of interest for diagnosing early-stage BD, and may offer insight in the mechanisms that drive conversion in the absence of familial predisposition. Findings from this study need to be verified through large-scale longitudinal imaging studies in naturalistic cohorts of MDD patients.

**Disclosure of Interest:**

None Declared

